# A global survey on occupational health services in selected international commission on occupational health (ICOH) member countries

**DOI:** 10.1186/s12889-017-4800-z

**Published:** 2017-10-05

**Authors:** Jorma Rantanen, Suvi Lehtinen, Antonio Valenti, Sergio Iavicoli

**Affiliations:** 1 International Commission on Occupational Health (ICOH), C/o Italian Workers’ Compensation Authority (INAIL), Department of Occupational and Environmental Medicine, Epidemiology and Hygiene, via Fontana Candida, 1, 00078 Rome, Monteporzio Catone Italy; 2 Department of Public Health/Occupational Health, University of Helsinki, PO Box 20 (Tukholmankatu 8 B), FI-00014 Helsinki, Finland; 3 Italian Workers’ Compensation Authority (INAIL), Department of Occupational and Environmental Medicine, Epidemiology and Hygiene, via Fontana Candida 1, 00078 Rome, Monte Porzio Catone Italy

**Keywords:** OHS, Legal basis, Capacity building, Infrastructures, Human resources, Coverage of services, Priorities in services

## Abstract

**Background:**

The United Nations General Assembly (UNGA), the International Labour Organization (ILO), the World Health Organization (WHO), the International Commission on Occupational Health (ICOH), and the European Union (EU) have encouraged countries to organize occupational health services (OHS) for all working people irrespective of the sector of economy, size of enterprise or mode of employment of the worker. The objective of this study was to survey the status of OHS in a sample of countries from all continents.

**Methods:**

A questionnaire focusing on the main aspects of OHS was developed on the basis of ILO Convention No. 161 and several other questionnaire surveys used in various target groups of OHS. The questionnaire was sent to 58 key informants: ICOH National Secretaries.

**Results:**

A total of 49 National Secretaries responded (response rate 84.5%), from countries that employ 70% of the total world labour force. The majority of the respondent countries, 67%, had drawn up an OHS policy and implement it with the help of national occupational safety and health (OSH) authorities, institutes of occupational health or respective bodies, universities, and professional associations. Multidisciplinary expert OHS resources were available in the majority (82%) of countries, but varied widely in quantitative terms. The average OHS coverage of workers was 24.8%, with wide variation between countries. In over two thirds (69%) of the countries, the content of services was mixed, consisting of preventive and curative services, and in 29% preventive only. OHS financing was organized according to a mixed model among 63% and by employers only among 33% of the respondents.

**Conclusions:**

The majority of countries have drawn up policies, strategies and programmes for OHS. The infrastructures and institutional and human resources for the implementation of strategies, however, remain insufficient in the majority of countries (implementation gap). Qualitatively, the content and multidisciplinary nature of OHS corresponds to international guidance, but the coverage, comprehensiveness and content of services remain largely incomplete due to a lack of infrastructure and shortage of multiprofessional human resources (capacity gap). The estimated coverage of services in the study group was low; only a quarter of the total employed population (coverage gap).

**Electronic supplementary material:**

The online version of this article (10.1186/s12889-017-4800-z) contains supplementary material, which is available to authorized users.

## Background

The Resolutions of International Organizations, the UN, ILO, WHO; ICOH, and the EU have called for their member countries to strengthen their OHS to better respond to the needs of the health and work ability of their working populations. Studies by the ILO and ISSA and the 27 EU Countries survey carried out in 2009 by the European Agency for Safety and Health at Work (EU-OSHA) reported that 61% of interviewed Europeans considered that the impact of the global financial crisis would lead to a deterioration in working conditions. The strategies and programmes of the International Organizations (ILO, WHO, UNDP, World Economic Forum), and most recently, UN Sustainable Development Goals (SDGs) Nos. 1, 3 and 8, have set targets and objectives for the provision of OHS for all working people [1–20].

The countries compete with each other through the quality and productivity of their workforces [10]. In addition to ensuring the health, safety and well-being of workers, a well-developed OHS system will also be in a key position to support the development of productivity and the prevention of productivity loss. This takes place through the prevention of sickness absenteeism and premature disability, the control of losses from occupational accidents and diseases, and through striving for longer working careers among the ageing working populations by promoting health, work ability and better work organization [10, 14, 20–22]. So far, most countries of the world have not organized OHS for the majority of workers [23, 24].

Information on the coverage, content, resources, governance, and financing of OHS in countries is scarce. A few studies by WHO Geneva, WHO-EURO as well as some individual researchers or research groups have examined research priorities in occupational health (OH) or OHS [22, 24–39].)

Some countries have drawn up National OHS profiles, which show the current OHS situation [38, 40–46]. Therefore, surveys are important in completing the data.

Only a few countries have established systematic national OHS statistics that would permit a detailed analysis of the status of OHS at national, regional and global levels [47, 48].

The most recent international call for the development of OHS for all is the United Nations (UN) Resolution on Sustainable Development Goals (SDGs 1, 3 and 8) [2].

The purpose of the present study was to survey, through key informants, the status of OHS at the national level and to estimate the global availability of OHS in view of the objectives of the international strategies and standards of the UN, ILO and the WHO [2, 4–6].

## Methods

### Questionnaire form

A questionnaire with 20 main questions, both structured and open in form, was developed using models of several other questionnaire surveys [34, 35, 49] used in various target OHS groups. Six senior experts from four countries tested the feasibility of the form by pre-filling. Seven questions had a space for clarifications (Questions 4, 5, 11, 12, 13, 14, 17). Two questions were open for listing national future priorities and providing information on changes and developments since the first ICOH survey in 2011. A space was given for complementary information and documentation, which several respondents provided in addition to their responses. The questionnaire was annexed with a list and explanations of the most relevant concepts and definitions used in the study. An advance information letter was sent to the study group. Complementary information provided by the respondents was combined for the analysis of results. The first survey of this kind was carried out among the ICOH National Secretaries in 2010–2011 [24]. The questions were similar to enable comparisons.

Table 1 describes the main domains of the survey.

**Table 1:** Main domains and themes of survey questions

Domain	Brief title	Question themes ^a^
Normative basis	Policy	• Ratification of ILO Conventions
	Strategy	• National policy and strategy
	Legislation and implementation	• OHS legislation • Steering and enforcement bodies • Implementation of ILO-OSH 2001
OHS resources	Institutions and human resources	• National institutions • Professional organizations • Human resources (physicians, nurses, hygienists, etc.) • Composition of OHS teams
Systems and infrastructures	Service provision models and service providers Workers’ access to OHS	• Service provision models • OHS coverage • Coverage of OHS’ support services • OHS for SMEs and the self-employed • Integration of OHS with PHC • Key actors in OHS
Substantive orientation and content of OHS	Principal orientation of OHS (preventive, curative, mix)	• List of OHS activities [49] • Application of BOHS activities [57] • Implementation of ILO-OSH Guideline [52]
OHS financing	Financing models	• Financing sources (employer, public budget, insurance, etc.)
Future developments	Priorities for OHS development	• 3–5 most important priorities for OHS development
Changes and developments since first survey	Developments in OHS since 2011	• Main changes in OHS system in any of the domains described above

^a^ This report describes the results of only a part of the study questions

### Study participants

The 2015 questionnaire was sent to all 58 ICOH National Secretaries on 4 March 2015 and three reminders were also sent out by 15 May 2015. A total of 49 forms were received by 31 May 2015. ICOH National Secretaries were selected as the key informants for this survey. The ICOH National Secretaries are elected and specially appointed by the ICOH President for three-year tenures from among ICOH members who are active in ICOH and known to have good contacts with the OH communities, stakeholders and actors in their countries. The responding National Secretaries were from countries covering 75% of the whole ICOH membership. The majority of respondents were affiliated with well-established national organizations, national institutes of occupational health and safety, universities, ministries, or national associations of occupational health (or a respective body).

### Statistical analysis

Pearson’s correlation coefficients were calculated in order to quantify the strengths of association and the direction of the relationship between ‘OHS coverage’ and the UNDP Human Development Index (HDI) and the World Economic Forum Competitiveness Index. Both analyses yielded a positive and statistically significant relation (*p* < 0.001) [12, 13].

## Results

### Response rates and geographical distribution

ICOH Regions were used as the basis for the geographical distribution of the countries (showing North American and Latin American countries separately and Oceania and Asia as two separate regions). The overall response rate was 84.5%. The highest absolute number of responding countries came from Europe, followed by Africa, Asia, and Latin America and the Caribbean, whereas the highest percentage rates were recorded for Africa and Oceania. The rates for all continents were 70% or over, except for that of North America, which was 33% (Table 2).

**Table 2:** Survey respondents

Continent	No. of responding countries	No. of ICOH member countries with NS	Response rate, %
Africa	11	11	100.0
Asia	8	9	88.9
Latin America and the Caribbean	7	10	70.0
North America	1	3	33.3
Europe	21	24	87.5
Oceania	1	1	100.0
Total	49	58	84.5

Since 2011, the number of responding countries has more than doubled in Africa and grown slightly in the Latin American Region, but decreased in Europe, North America and Asia. A total of 13 countries that responded in 2011, and represented 4.9% of the total global workforce, did not respond in 2015. In addition, 15 new respondents were obtained in 2015, employing 281.1 million workers (8.3% of the world total). Thus, the total study base grew from 1.973 billion workers in 2008 to 2.356 billion in 2014, i.e. by 19.5%. In sum, the respondents of the 2015 survey came from countries in which 70% of the workers of the world were working [50].

### Normative basis

#### Ratification of international instruments

A total of 22 respondent countries (45%) have ratified ILO Convention No. 155 (33% of all the 66 countries that have ratified this Convention), 14 countries (28.5% of the respondents) have ratified ILO Convention No. 161 (42% of the total of 33 ratifiers), and 15 countries (30.6%) have ratified Convention No. 187 (37% of the total of 41ratifiers) [4, 14, 51]. Thus, collectively, about a third of all ratifications of the three key OSH Conventions have taken place in the respondent countries. The ratifications of Convention No. 161 are recorded from Africa, Europe, Latin and North America, but none from Asia.

Although only less than one third of the respondent countries have ratified ILO Convention No. 161 on Occupational Health Services, twice as many have a policy and strategy for OHS in place (Table 3).

**Table 3:** Normative basis and governance of OHS in respondent countries

Normative basis and governance	Number of countries	%
Ratification of ILO Convention No. 161	14	29
Policy on OHS	33	67
Strategy on OHS	32	65
Governance		
- Ministry of Health (MOH)	10	20
- Ministry of Labour (MOL)	18	37
- Joint (MOH-MOL)	18	37
- Other	3	6

#### Formally adopted national OHS policy and strategy

ILO Convention No. 187 [14] and the WHO Global Plan of Action on Workers’ Health [6] call for a national policy framework and strategy on OSH and workers’ health. The majority of the countries (33/49, 67% of respondents) had an officially adopted OHS policy, or OHS was dealt with as a part of the OSH policy. The policy principles were also sometimes provided in the explanatory statement for labour legislation. In 69% of the respondent countries, the OHS policy was endorsed at a high political level, by the parliament, government as a whole, or by the responsible ministry, as recommended by the ILO. A total of 79% of the countries that had ratified ILO Convention No. 161 reported having a national OHS policy, whereas the respective percentage among the non-ratifiers was 63%.

In all, 32 countries (65%) had an independent national OHS strategy: nine of the ratifying countries and 23 of the non-ratifying countries.

#### Governance

Several OSH laws include provisions for collaboration and joint decision by the Ministry of Labour (MOL) and the Ministry of Health (MOH). Joint steering by the MOH and the MOL (with their subordinated government agencies) was reported by 37% of the respondents as the model for the governance and implementation of OHS. An equal percentage was reported for governance by the MOL alone. The MOH alone held governance in 20% of countries.

#### ILO-OSH management systems

A total of 49% of respondent countries had implemented the ILO-OSH Management System [52], an instrument that provides authorities and workplaces with a systematic approach to the organization of OSH activities at different levels of the system. Social partners employers and workers, also played an important role in OHS governance in the majority (61% and 67%, respectively) of the countries.

### OHS resources

#### National institutions for occupational health and occupational safety and health

Twenty-seven countries (55% of respondents) had either a national institute of occupational health or a respective unit in the jurisdiction of the relevant ministry. In some countries (e.g. Croatia), the responsibilities and activities of the national institute were delegated to several institutions. In others, a part or all of the activities of a national institute were carried out by the universities.

#### Professional organizations and associations

In most of the respondent countries, occupational health physicians (OHPs) had an association of their own (43 countries, 88% of respondents). Similarly, 30 countries (61% of respondents) had associations for occupational hygienists, 28 for ergonomists (57%) and 33 (67%) for safety engineers. The number of the countries that had professional associations of occupational health nurses (OHNs) was smaller, at 22 (45%). Twelve countries reported having an association of occupational psychologists (24%). Some of the countries also had associations for other professionals in the OSH field, for example, work organization experts.

#### Human resources for OHS

Table 4 shows the availability of various OHS professional groups in the respondent countries.

**Table 4:** Human resources for OHS

OHS personnel	Availability in the country		Data available on the numbers		Numbers of OHS personnel	% of total
	n	%	n	%		
Occupational health physicians	49	100	43	88	143,522	35
Occupational health nurses	34	69	29	59	75,365	18
Occupational hygienists	33	67	29	59	35,290	9
Safety engineers	40	85	28	57	149,147	35
Ergonomists/occupational physiotherapists	31	63	24	49	9753	2
Occupational psychologists	25	31	19	39	2953	1
Total					416,030	

OHPs and safety engineers were the largest expert groups, followed by OHNs and occupational hygienists. The resources of psychologists and ergonomists were very low. Multidisciplinary OHS teams with four to seven expert categories were reported by 40 countries (82%) and monodisciplinary OHS teams (fewer than four expert categories) in nine countries (18%); The multidisciplinary expert resources were available in 70% of the countries with MOH model, 94% with MOL model and in 78% of the countries with joint model. This is in line with the occurrence of the ‘comprehensive content’ and ‘ILO standard content’ of OHS, which both require multidisciplinary staff for implementation.

#### Composition of OHS teams

One of the basic principles in the development of OHS according to ILO Convention No. 161 is to organize services in a multidisciplinary team, instead of using individual experts. An optimal team would comprise an OHP, OHN, occupational hygienist, ergonomist, safety engineer, occupational psychologist, and other disciplines closely related to OH. The competence profiles of OHS teams in most countries were multidisciplinary, as proposed by the ILO. In eight countries, seven, and in 11 countries, six disciplines were available. Some of the expertise was only occasionally available, whereas the physicians’ and nurses’ services were more regularly available. We defined the teams with health personnel only, as monodisciplinary.

Data on the numbers of physicians working in OH were available in 43 countries (88% of respondents), on nurses and on occupational hygienists working in OH in 29 countries (59%, respectively),, and safety engineers in 28 countries (57%). Most countries reported their total numbers of experts, but not the full-time equivalents. Not all the reported professionals were necessarily specialists. The density of experts, i.e. the average number of served workers per one expert, for example, an OHP, varied substantially between the responding countries; the average density in the whole responding material being one physician per 16,416 workers. Table 4 presents the total numbers of reported OHS experts in the respondent countries. The densities of safety engineers were the highest, followed by OHPs.

The densities of experts in relation to the total employed population varied in orders of magnitude. For example, the highest densities of OH experts were reported in Finland, one OHP/1234 employees and one OHN/1045 employees, and Italy had one OHP/2478 employees. The lowest densities were recorded in developing countries, India had one OHP/66581 workers in the total labour force, and some African countries had one OHP/1–4.5 million employees. If all the OHS experts of the respondent countries are counted together, the average density amounts to one OH expert/5663 workers. The total work-time input for OHS by non-medical personnel is, however, estimated to be substantially lower than the full-time equivalent.

#### Training of OHS personnel

The level of training of OHS personnel was measured using the availability of various specialties in the OHS teams. Forty-four countries (90% of respondents) had a specialty in occupational medicine or occupational health, and special training for OHNs was organized in 21 countries (43% of respondents). Specialist training for occupational hygienists was organized in 28 countries (57% of respondents). Several countries provided shorter training for OH experts who were not specialists. Training was provided by university medical faculties for basic curricula of OHPs and by nursing schools, universities and polytechnics for OHNs. The professional associations contributed together with universities to complementary training. Professional associations, medical chambers or specialty boards on the basis of formal authorization were responsible for examining and granting specialties and diplomas to trainees.

A majority of physicians in OHS did not have specialist training in occupational health/occupational medicine, but had some training in OH. Among the nurses, a few months to one-year training in OH was common, while proper specialist training of three to 4 years was rare. The ratio of the numbers of physicians to nurses working in OH was 1.9.

The experiences of integrating OHS with primary health care (PHC) identified a need for training in OH for PHC personnel as a critical prerequisite for the provision of basic OHS in PHC units [53]. Some countries commented on an urgent need for OH training of OHS providers within the PHC system in particular.

### OHS systems and infrastructures

#### Service provision models for OHS

Table 5 presents the service provision models utilized in the respondent countries.

**Table 5:** Availability of different service provision models used in respondent countries

Provision model	Number of countries	% of respondents
Big industry in-plant service	45	92
Group service	32	65
PHC units or other PH service	37	76
Hospital polyclinics	26	53
Private services	41	84
Other model	15	31
Number of respondents	49	

Most of the countries had organized service provision through multiple models (Table 5). A total of 92% of the respondent countries utilized the big industry model, in which a company-specific OHS unit provides services for the employees of a big company, usually with 500 employees or more. The private services model was used by 84% of the respondents. A total of 76% of countries also utilized services provided by PHC units as one service provision model. Group services, organized jointly by several, usually medium-sized or small companies, were utilized by 65% of the respondents.

#### Coverage of OHS

Coverage means the percentage of workers of the total workforce with access to OHS.

**Fig. 1:**
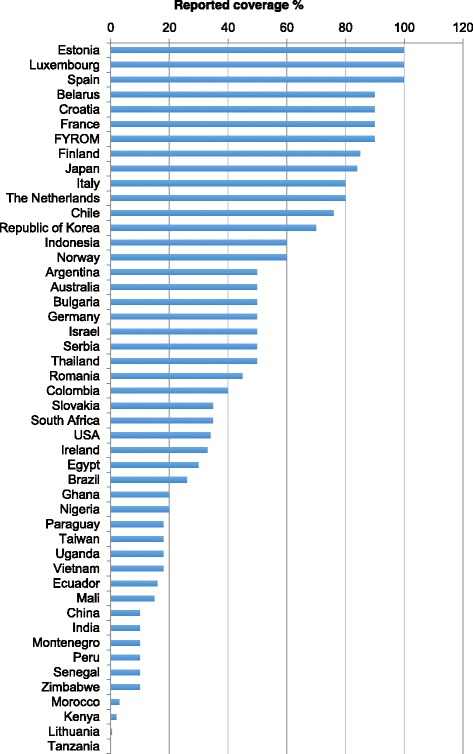
Estimated coverage of OHS reported in 48 countries

OHS coverage in the countries varied widely (0.5%–100%). MOH governance showed higher coverage (58%) than OHS in MOL (21%) or joint governance (20%). A total of 31% of the respondent countries had more than 50% coverage of employees, but the majority had a lower percentage or did not provide data on their coverage (Fig. 1). In 2014, the emerging economies with large working populations; China, India and Brazil, together employed a total of 68% of the workers of the surveyed worker population. Coverage was 26% in Brazil, 10% in China [54] and below 10% in India [55]. Many of the countries with a high coverage (75% to 97%), for example Croatia, Finland, FYR Macedonia, and the Netherlands are relatively small, with only a minor impact on the global coverage. However, coverages exceeding 75% were also reported in bigger countries such as France, Italy and Japan.

A rough estimate of the population with access to some kind of OHS among the worker population of the respondent countries was approximately 585 million, making the average coverage of the total employed population 24.8%. The majority (1.77 billion) of the 2.36 billion economically active workers (75.2%) in the surveyed countries, however, did not have access to OHS.

#### Integration of OHS with PHC

WHO has recently emphasized the revitalization of PHC and the integration of health services at the grassroots level [56]. In almost half, 47%, of the respondent countries OHS was fully or partly integrated with PHC. The level of integration varied from assignment of OHS tasks to PHC personnel or to organization of OHS within the PHC units as a separate activity (e.g. Croatia, Finland, FYR Macedonia).

A total of 27 responding countries (55%) reported that they had introduced the Basic Occupational Health Services (BOHS) approach [57] in order to extend the coverage of OHS to uncovered sectors and workers. Of these, 12 countries had organized BOHS as a separate occupational health service, and an additional 17 respondents reported having integrated BOHS with their PHC.

The likelihood of OHS being integrated with PHC was more common under MOH governance.

### OHS contents and activities

The substantive content of OHS is guided by several international instruments and numerous national guidelines [4, 49, 58–61]. In 34 respondent countries (69%), the main orientation was mixed, combining both preventive and curative activities, and in 14 countries (29%) preventive only. One of the respondents provided curative activities only. Half of the services provided under MOH governance were preventive only, while the respective share in the MOL- and joint MOH-MOL governance models was one-fifth.

The impact of the ratification of Convention No. 161 on the content of OHS was analysed. The comprehensive content was most prevalent (51% of total) and equally represented among the ratifiers and non-ratifiers. Second was the ‘ILO standard content’ (31% of total).

Tmain orientation of OHS (preventive/curative) was different depending on the governance model. As the preventive only and mixed (preventive + curative) were equally represented in the MOH model, the vast majority (75%) of MOL and joint models favoured the mixed content.

The comprehensiveness of OHS was measured by the number of different types of activities included in the OHS programme (Table 6). Services with 13–14 activities including prevention, promotion, curative care, rehabilitation, information, and training and education (60) were considered comprehensive, and services with 10–12 activities were considered ‘ILO standard content’, corresponding roughly to ILO Convention No. 161 provisions, and services with 1–9 activities were classified as limited.

**Table 6:** Occurrence of various OHS activities

Activities	No. of countries	%
Orientation and planning	39	80
Surveillance of work environment	46	94
Surveillance of workers’ health	46	94
Assessment of health and safety risks	44	90
Information and education	45	92
Preventive actions	42	86
Prevention of accidents	42	86
First aid	42	86
Diagnosis of occupational and work-related diseases	42	86
Promotion of health and work ability	43	88
General health care	30	61
Curative care and rehabilitation	30	61
Record-keeping	43	88
Evaluation and auditing	33	67

A higher percentage of countries with MOL governance (72%) followed the comprehensive content model. MOH-governed OHS had such wide content in 50% of the countries, whereas it in the joint MOH + MOL governance was reported in 39%.

The multidisciplinary content of services was common in the responses; over 82% of respondents reported a total of 10 or more different OHS activities, including prevention, risk assessment, surveillance of work environment and workers’ health, health education and information, diagnosis of occupational diseases, and prevention of accidents. The provision of such content requires multidisciplinary OHS staff. The vast majority, 82% of the respondents, used four or more expert OHS categories.

### Financing of OHS

The ILO principles stipulate that the primary responsibility for financing OHS rests on the employer. This responsibility can be met by either direct financing or through employer payment of insurance premiums (35% of respondents). Most respondent countries used multiple sources of funding (63% of respondents) (Table 7).

**Table 7:** Financing models

Financing mechanism	No. of countries	%
Employers only	16	33
Public sector only	–	–
OSH Insurance	1	2
Special Insurance	–	–
General Social Insurance	1	2
Combination of some of the above	31	63
Other		

None of the countries financed OHS from public funds only. The big industries in particular often financed on their own.

### Future priorities

**Fig. 2:**
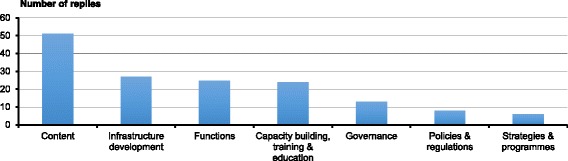
Future OHS development priorities of the responding countries

The respondents were asked to mention 3–5 priorities for future development of OHS. A total of 44 countries (90%) responded to the question of future priorities and gave a total of 154 priority items. Seven groups of priorities were recognized, the most common being:

1. The development of the content of OHS to keep abreast with changing work life
2. Infrastructure development to improve the provision of OHS
3. Development of OHS functions, such as mainstreaming of OHS and developing information systems, and
4. Capacity building, particularly training of OHPs, OHNs and other OH experts, as well as the integration of OHS elements into the curricula of other experts (such as engineers) (Fig. 2).

### OHS summary profiles and development indices

**Fig. 3:**
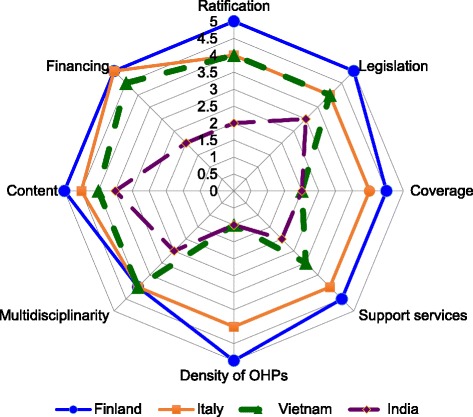
Arbitrary profiles on main domains of four countries drawn up on the basis of the survey. (Scaling criteria provided in Additional file 1)

Figure 3 represents an example of an eight-domain profile, using data from four countries in this survey [38, 40, 62, 63]. In general, the differences in the development stage of OHS are demonstrated by several parameters.

**Fig. 4:**
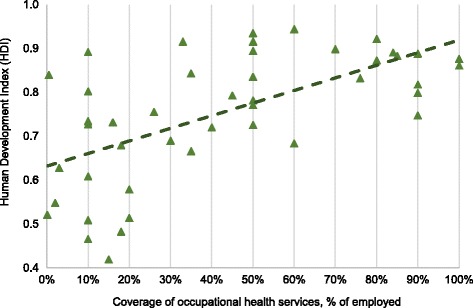
Correlation between coverage of OHS and the UNDP Human Development Index, 2014 (HDI) (*R* = 0.62, *p* < 0.001) [12]

**Fig. 5:**
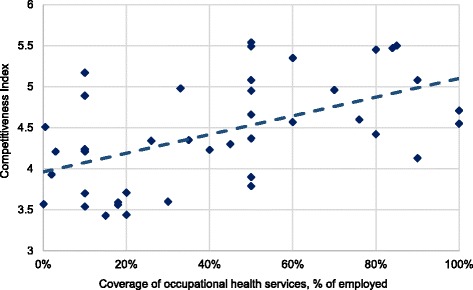
Correlation between coverage of OHS and the World Economic Forum Competitiveness Index, 2014 (*R* = 0.54, p < 0.001) [13]

The reported OHS coverage figures were correlated with the UNDP HDI and the World Economy Forum competitiveness indices [12, 13] (Figs. 4 and 5). The two indices correlated positively with the growing coverage of OHS.

## Discussion

### Representativeness of the study

Key informant surveys have been widely used in the research of health services systems [64–67]. The present study used the definition of the key informant by Parsons et al. [67].

This survey can be mainly considered as a qualitative study of OHS among key informants (ICOH National Secretaries) in countries with organized ICOH-related professional activities.

The response rate of the ICOH national secretaries was 84.5%. Collectively, the respondent countries represented roughly 75% of total ICOH membership.

### Limitations of the method

The survey questions were designed by examining several other surveys and their questionnaire verbiage as a starting point [34–37, 49]. The low rate of ‘not available’ responses speaks in general for the good feasibility of the questionnaire form.

Only one key informant per country was available for the survey, which may limit the scope of information provided in the responses. The guidance given for respondents on consultation at the national level may have compensated for this to a certain extent.

Three factors may cause positive bias in the representativeness of the current study. First, it is assumed that the proxy respondents interpreted the situation more positively than the constituents [67], for example in the assessment of OHS in SMEs and among the self-employed. Second, the larger size of the OH community with national secretaries is assumed to reflect a relatively well developed OHS system. Third, the responses are mainly qualitative in principle. For example, as the comprehensive content of OHS is reported by high numbers of countries, the result cannot be interpreted as nation-wide coverage of comprehensive OHS. However, an identifiable part of OHS may have such content. Therefore, conclusions regarding the average global situation on the basis of the current study should be drawn with caution.

However, the base of the present study was unique, due to well-informed expert participants. The lack of reliable registries and statistics in most countries affects the availability of quantitative data.

### Normative basis

The key international instruments provide guidance for the development of the OHS system, contents and good practices [4–6, 58–60, 68, 69],

ILO Convention No. 161 calls for drawing up a national OHS policy and programme. The survey indicates that the respondents are well informed of the requirements of the international instruments and that the instruments’ impact was more wide ranging than the extent of formal ratifications. OH policies were more widespread than strategies and programmes. This reflects the challenges of practically implementing policies in countries (implementation gap). It seems that ratification is not a prerequisite for an OH policy and strategy, and that the ILO instruments’ guidance is used without ratification.

In addition to ministries and authorities, social partners, employers and workers play an important role in the Government Advisory Bodies and in practical implementation. In such a multi-stakeholder setting, it is important that all the partners have equal access to information on OHS. An OHS profile provides equal information for all partners in the governance of OHS. Such profiles reveal both the strengths of the system and its needs for development. ILO and WHO encourage countries to draw up national OSH and OHS profiles [3, 38, 40, 46].

The majority of the respondent countries stipulate, through OSH legislation, that employers are obliged to organize OHS for workers. An independent stand-alone law on OHS exists only in Finland, whereas several countries, for example, Italy and Thailand, authorize health centres by law to provide OHS.

### Human resources for OHS

Limited human resources for OHS constitute an obstacle in the achievement of the universal provision of services (capacity gap). The most important obstacles in the provision of services for small-scale enterprises, the self-employed and informal sector workers are the lack of service infrastructures and shortage of trained OHS personnel. A total of 49 countries reported on the availability of OHPs. A formal specialty in occupational medicine/occupational health is available in 90% of the respondent countries, but the absolute numbers of the specialists are, in the experience of the authors, very low. OHNs were available in only 34 respondent countries. In many countries, the profession of OHN is non-existent, while in some countries they constitute the most important expert group of OHS.

The absolute numbers of personnel in various expert categories, however, do not enable the organization of multidisciplinary services to the extent reported, due to the low availability of ergonomists and occupational psychologists.

A minimum density of OH experts, one OHP and two OHNs per 5000 workers, has been proposed on the basis of practical experience [57]. The present average density of physicians and nurses working in OH in the respondent countries is one expert per 10,764 workers. Covering the capacity gap in the respondent countries would require doubling present resources, and filling the gap in the whole world would mean a three-fold number of OHPs and particularly OHNs. Instead of the recommended ratio of 0.5, the present survey found the ratio between physicians and nurses in OHS to be 1.9.

In some countries, such as Croatia, the former Yugoslav Republic of Macedonia and Serbia [35], physicians providing OHS in PHC units are specialists in occupational medicine. In Finland, a third of OHPs in municipal OHS are OH specialists and must have special training in OHS [47], whereas 51% of physicians in big industry services are specialists.

To meet the international recommendations for multidisciplinary and comprehensive services, numbers of other OH experts are insufficient (occupational hygienists, psychologists, ergonomists).

The UN High-level Commission on Health, Employment and Economic Growth (2016) has proposed a target of 40 million new health and social workers by the year 2030 [70]. Reaching full coverage of OHS for all working people would require 1.5% of this resource for OHS (0.6 million).

### OHS systems and infrastructures

The key aspects of OHS infrastructures are service provision capacity, coverage and contacts with workers, employers and workplaces.

#### Service provision models

A total of 90% of respondents have three or more service provision models available; the big industry, private centre model and the PHC centre model being the most common. Thus, the availability of an alternative service provision model enables services for several different types of enterprise. Further delegation of OH to PHC staff or general practitioners (GPs) has been proposed [53, 57]. This is, however, unrealistic, as WHO reports a 40-million shortage of health personnel in general in the world by 2030, particularly in PHC. Even in well-developed health systems such as that in Finland, PHC workers report high workloads and psychological stress [70–72].

#### OHS coverage

Question 16 in the current survey elicited the coverage of the workers as a percentage of the total employed population, i.e. the proportion of the workers of the total workforce with access to OHS.

Wilson et al. found a statistically significant association between the ratification of relevant ILO Conventions (Convention No. 161 in particular) and lower occupational accident fatality rates than in non-ratifier countries [73]. Ratification may facilitate OSH programmes in general, including OHS. However, average coverage in the world is still low, as was the estimated global coverage of OHS in the present survey, which was 18.8%.

The increase in coverage in the present survey was due to the higher average coverage of the new respondents compared with that of the 2011 survey, and was related to the change in the study base.

In view of the requirements of the International Instruments [4, 5, 74], and often of the law applicable in the countries, the OHS coverage of workers in the surveyed countries is insufficient, with only a few exceptions (coverage gap). The coverage gap is seen particularly among workers in small-scale enterprises, the self-employed, agriculture, and the informal sector. ILO Convention No. 161 and the WHO Global Strategy on Occupational Health for All request the universal provision of OHS for all working people. As on average 80% of the total working population in the world do not have access to services, special and intensive actions are needed for expanding the coverage of OHS.

In order to ensure the widest possible coverage of OHS, a new BOHS approach was introduced as a joint priority of development and collaboration between ILO, WHO and ICOH [7, 57]. The UN SDGs now emphasize the need to provide OHS for all (SDG 3) [2] by scaling up basic and specialized OHS.

Poor OHS registration and statistics may affect the recognition of the real needs of OHS and may lead to inaccuracies in coverage estimates. In a few countries, for example, Finland, France, Japan, the Netherlands, which have a high coverage of above 80% and well-established registration or survey-based OHS statistics, the available human resources and coverage figures match well, reflecting the true situation. The gap found among the SMEs, the self-employed and the informal sector is universal, particularly in emerging and developing economies. In many countries, the available number of OH experts is so limited that the level of officially reported coverage is impossible to achieve in practice.

### Content of OHS

The most common orientation of OHS among the respondents was mixed (preventive and curative). Question 13 listed 14 OHS activities of which half were preventive according to ILO Recommendation No. 171 and the 1990 WHO/EURO recommendations [49, 58]. The multidisciplinary content of services was common in the replies; over 82% of respondents reported a total of 10 or more different OHS activities, including prevention, risk assessment, surveillance of the work environment and workers’ health, health education and information, diagnosis of occupational diseases, and prevention of accidents. The provision of such content requires multidisciplinary OHS staff. A total of 82% of respondents used four or more expert OHS categories. However, two biases are likely to affect the results of the present study. First, in some countries, OHS activities mainly focus on workers’ health examinations that are considered primarily preventive with minimal or no workplace-oriented activities. Second, the qualitative information does not describe the national coverage of comprehensive multidisciplinary services. As the numbers of the experts, such as ergonomists and occupational psychologists in the survey material are several times lower than the numbers of OHPs and OHNs, the average availability of preventive and comprehensive services remains limited. A considerable ‘gap’ in real content prevails in many countries.

### Financing

In the majority of the countries, arrangements for OHS financing are based on mixed employer and insurance funding. This fits the well-organized sectors of work life, but most of the workers in the coverage gap are employed in less organized settings, often with no social protection or insurance, and a majority has no formal employment contract and thus no employer. Organizing funding for OHS in such sectors needs public interventions either through direct action by the government or through public social insurance. The social insurance model may provide more long-term stability and sustainability for services. Some countries have organized insurance-based funding for OHS on the principle of solidarity, i.e. the sectors and enterprises that are able to contribute pay slightly higher premiums than their mathematical share to cover the costs of the non-contributing sectors [35].

In Thailand, the BOHS services provided by PHC units are financed from public sources. In the Republic of Korea, publicly financed services for small enterprises are provided by special OH centres. In Croatia, an OSH insurance for health protection at work covers all the costs of OHS using premiums paid by employers.

OHS coverage can be assessed in view of the HDI and economic competitiveness [12, 13, 75] (see Figs. 4 and 5). The financial loss from occupational accidents and diseases has been estimated to be 4%–5.9% of GDP [75–78], corresponding to about half of the total health budgets of many countries. Although data are not available, the loss among small enterprises and the self-employed may even elevate the loss estimate. Evidence from industrialized countries show that OSH has a positive impact on the national economy [79, 80]. In the present survey, investment in OHS does not negatively affect the competitiveness or HDI of the countries.

The UN High-level Commission on Health, Employment and Economic Growth [70] has estimated the return on investment (ROI) in health to be 9:1 and a one-year increase in life expectancy, raising GDP by 4%. We can assume that the improvement of health and life expectancy of the working population plays a large part in this positive impact. For example, providing good OHS for the health sector would add to the existing health workers’ input by 19%–20%, without adding new personnel [70, 81].

### Future priorities

The respondents identified a great deal of priorities for future OHS development (Fig. 2). The priorities were principally directed towards the content, infrastructures, functions, and capacities of OHS, i.e. towards strengthening implementation rather than towards the policy or strategy already available in the majority of the countries. The responses suggest that the countries have recognized the gaps in human resources, content and coverage, and seek solutions to these, which are all prerequisites for the implementation of OHS policies and strategies.

## Conclusions

The ICOH National Secretaries can serve as key expert informants on the OHS in their countries.

The international instruments remain valid and provide good guidance for the development of OHS at the national level. The UN strategy for SDGs further emphasizes the needs for ratification and implementation. The challenges of globalizing work life need OHS with comprehensive content and multidisciplinary human resources. Two thirds of the surveyed countries have both a qualitative (lack of multidisciplinary experts) and quantitative shortage of expert human resources (capacity gap), which is an obstacle to the achievement of full OHS coverage. Providing universal access to OHS for all working people is considered to support the socio-economic development of countries. Services are also needed for the prevention of the annual 2.3 million work-related fatalities and major economic loss.

In two thirds of the respondent countries, a wide gap in the implementation of policies into practice leaves the majority of workers without access to OHS. This implementation gap, found mainly among SMEs, the self-employed and informal sector workers, is associated with the limited availability of the necessary infrastructures, and low OHS coverage.

These shortages also affect the content of OHS (content gap). The estimated global coverage of OHS is less than 18.8% (coverage gap). In order to expand coverage, almost half of the respondent countries have undertaken actions to integrate OHS with PHC, and 55% have introduced the basic OHS approach in their OHS system.

The development of services for workers’ health needs national information and statistics on OHS, including policies; institutional, human and financial resources; and the structures, coverage, contents, and activities of services in the countries.

The countries have proficiently recognized the future priorities for the development of their OHS systems, and emphasize the need to develop the prerequisites for the practical implementation of OHS. In order to achieve the UN SDGs, the UN High-level Commission has proposed a target of 40 million new health and social workers by 2030. Meeting the SDGs for workers’ health would require about 0.6 million (1.5% of the proposed 40 million) OH workers by 2030.

The development of OHS throughout the world is a critical prerequisite for the achievement of the UN SDGs, particularly SDGs 1, 3 and 8 [2, 78, 82].

## Additional file

Additional file 1: Criteria for OHS Index. (DOCX 17 kb)

## Abbreviations

BOHS: Basic occupational health services
EU: European Union
EU-OSHA: European agency for safety and health at work
GDP: Gross domestic product
GP: General practitioner
HDI: Human development index
ICOH: International commission on occupational health
ILO: International Labour Organization
ISSA: International Social Security Association
MOH: Ministry of health
MOL: Ministry of labour
OH: Occupational health
OHNs: Occupational health nurses
OHPs: Occupational health physicians
OHS: Occupational health services
OSH: Occupational safety and health
PHC: Primary health care
ROI: Return on investment
SDGs: Sustainable development goals
SME: Small and medium-sized enterprises
UN: United Nations
UNDP: United Nations development programme
UNGA: United Nations general assembly
WHO: World Health Organization

## References

[1] United Nations General Assembly. Keeping the promise: united to achieve the Millennium Development Goals (A/RES/65/1). 2010. http://www.un.org/en/mdg/summit2010/pdf/outcome_documentN1051260.pdf. Accessed 18 Nov 2016.

[2] United Nations. Sustainable Development Goals: 17 goals to transform our world. http://www.un.org/sustainabledevelopment/sustainable-development-goals/. Accessed 18 Nov 2016.

[3] International Labour Organization. Global Strategy on Occupational Safety and Health. Conclusions adopted by the International Labour Conference at its 91st Session. Geneva: ILO Publications; 2003. http://www.ilo.org/safework/info/publications/WCMS_107535/lang%2D-en/index.htm. Accessed 18 Nov 2016.

[4] International Labour Organization. Convention No. 161 concerning Occupational Health Services. Geneva: International Labour Conference 71st session; 1985. http://www.ilo.org/ilolex/english/convdisp1.htm. Accessed 18 Nov 2016.

[5] World Health Organization. WHO Global Strategy on Occupational Health for All. The Way to Health at Work. Geneva: WHO; 1995. http://www.who.int/occupational_health/globstrategy/en/. Accessed 18 Nov 2016.

[6] World Health Organization. WHA Resolution 60.26. Global Plan of Action on Workers' Health 2008–2017. Geneva: WHO; 2007. http://www.who.int/occupational_health/WHO_health_assembly_en_web.pdf. Accessed 18 Nov 2016.

[7] International Labour Organization. Report of the 13th Joint ILO/WHO Committee on Occupational Health. Geneva: ILO; 2003. http://www.ilo.org/safework/info/publications/WCMS_110478/lang%2D-en/index.htm. Accessed 18 Nov 2016.

[8] International Commission on Occupational Health. Centennial Declaration of the International Commission on Occupational Health. Milan: ICOH; 2006. http://www.icohweb.org/site_new/multimedia/core_documents/pdf/centennial_declaration.pdf. Accessed 18 Nov 2016. 17219772

[9] International Commission on Occupational Health. Seoul Statement on Occupational Health Services. Seoul: ICOH; 2015. http://www.icohweb.org/site/multimedia/core_documents/pdf/ICOH2015_Seoul_Statement.pdf. Accessed 29 Nov 2016.

[10] Commission of the European Communities. Improving quality and productivity at work: Community strategy 2007-2012 on health and safety at work. Communication from the Commission to the European Parliament, the Council, the European Economic and Social Committee and the Committee of the Regions COM (2007) 62 final. Brussels: Commission of the European Communities; 2007. http://eur-lex.europa.eu/LexUriServ/LexUriServ.do?uri=COM:2007:0062:FIN:EN:PDF. Accessed 29 Nov 2016.

[11] European Commission. Strategic Framework on Safety and Health at Work 2014-2020. Communication from the Commission to the European Parliament, the Council, the European Economic and Social Committee and the Committee of the Regions COM(2014) 332 final. Brussels: European Commission; 2014. http://ec.europa.eu/social/main.jsp?catId=151. Accessed 30 Nov 2016.

[12] United Nations Development Programme. Human Development Report 2014. Sustaining Human Progress: Reducing Vulnerabilities and Building Resilience. New York: United Nations Development Programme; 2014. http://hdr.undp.org/sites/default/files/hdr14-report-en-1.pdf. Accessed 29 Nov 2016.

[13] World Economic Forum. World Economy Forum. The Global Competitiveness Report 2014-2015. Full data edition. Schwab K, editor. Geneva: World Economic Forum; 2014. http://www3.weforum.org/docs/WEF_GlobalCompetitivenessReport_2014-15.pdf. Accessed 29 Nov 2016.

[14] International Labour Organization. Convention No. 187 concerning the Promotional Framework for Occupational Safety and Health. Geneva: International Labour Conference 95th session; 2006. http://www.ilo.org/dyn/normlex/en/f?p=NORMLEXPUB:12100:0::NO::P12100_ILO_CODE:C187. Accessed 2 Dec 2016.

[15] World Health Organization, International Labour Organization. WHO-ILO Joint Effort on Occupational Health and Safety in Africa. Meeting Report Occupational health for workers in the informal sector Pretoria, South Africa 16–20 October 2000. http://www.who.int/occupational_health/publications/afrpretoria/en/index.html. Accessed 2 Dec 2016.

[16] International Labour Organization. World Commission on the Social Dimension of Globalization. A fair globalization: Creating opportunities for all. Geneva: ILO; 2004. http://www.ilo.org/public/english/wcsdg/docs/report.pdf. Accessed 2 Dec 2016.

[17] European Agency for Safety and Health at Work. European opinion poll on occupational safety and health. 2012. http://osha.europa.eu/en/statistics/eu-poll/slides/Package_EU27.pdf. Accessed 2 Dec 2016.

[18] International Labour Organization. Emerging risks and new patterns of prevention in a changing world of work. Geneva: ILO; 2010. http://www.ilo.org/wcmsp5/groups/public/-%2D-ed_protect/-%2D-protrav/-%2D-safework/documents/publication/wcms_123653.pdf. Accessed 2 Dec 2016.

[19] International Labour Organization. Health and life at work: A basic human right. Geneva: International Labour Office; 2009. http://www.ilo.org/public/portugue/region/eurpro/lisbon/pdf/28abril_09_en.pdf. Accessed 3 Dec 2016.

[20] International Social Security Association (2010). Dynamic Social Security: Securing social stability and economic development. Developments and Trends Global Report 2010.

[21] Meier V, Werding M. Ageing and the Welfare State: Securing Sustainability. Munich: CESIFO Working Paper No. 2916; 2010. http://www.cesifo-group.de/DocDL/cesifo1_wp2916.pdf. Accessed 3 Dec 2016.

[22] Tompa E, Banting KG, Sharpe A, St-Hilaire F (2002). The impact of health on productivity: Empirical evidence and policy implications. The review of economic performance and social progress.

[23] Elgstrand K, Lehtinen S, Rantanen J, Elgstrand K, Liesivuori J, Peurala M (2005). Do occupational health services really exist?. Challenges for occupational health services in the regions.

[24] Rantanen J, Lehtinen S, Iavicoli S (2013). Occupational health services in selected International Commission on Occupational Health (ICOH) member countries. Scand J Work Environ Health.

[25] Eakin JM, Cava M, Smith TF (2001). From Theory to Practice: A Determinants Approach to WHP in Small Businesses. Health Promot Pract.

[26] Martin A, Sanderson K, Cocker F (2009). Meta-analysis of the effects of health promotion intervention in the workplace on depression and anxiety symptoms. Scand J Work Environ Health.

[27] Goldgruber J, Ahrens D (2010). Effectiveness of WHP and primary prevention interventions: a review. J Public Health.

[28] Rantanen J, Kim R (2012). Situation analysis and recommendations for stewardship on workplace health promotion in Poland.

[29] European Agency for Safety and Health at Work. Priorities for occupational safety and health research in Europe: 2013-2020. Luxembourg: Publications Office of the European Union; 2013. https://osha.europa.eu/en/tools-and-publications/publications/reports/priorities-for-occupational-safety-and-health-research-in-europe-2013-2020/view/. Accessed 7 Dec 2016.

[30] Iavicoli S, Rondinone BM, Marinaccio A, Fingerhut M (2006). Research Priorities in Occupational Safety and Health: A Review. Ind Health.

[31] Iavicoli S, Marinaccio A, Vonesch N, Ursini CL, Grandi C, Palmi S (2001). Research priorities in occupational health in Italy. Occup Environ Med.

[32] Sadhra S, Beach JR, Aw TC, Sheikh-Ahmed K (2001). Occupational health research priorities in Malaysia: a Delphi study. Occup Environ Med.

[33] Van der Beek AJ, Frings-Dresen MH, van Dijk FJ, Houtman IL (1997). Priorities in occupational health research: a Delphi study in The Netherlands. Occup Environ Med.

[34] World Health Organization. Global Plan of Action on Workers’ Health (2008-2017): Baseline for Implementation Global Country Survey 2008/2009. Geneva: WHO; 2013. http://www.who.int/occupational_health/who_workers_health_web.pdf. Accessed 8 December 2016.

[35] Rantanen J (2010). Occupational health systems in South-East Europe.

[36] Hämäläinen R-M, Husman K, Räsänen K, Westerholm P, Rantanen J, Lehtinen S, Räsänen K, Husman K, Rantanen J (2001). Survey of the Quality and Effectiveness of Occupational health services in the European Union, Norway and Switzerland. People and Work. Research Reports 45.

[37] Westerholm P, Walters D, editors. Supporting Health at Work - International Perspectives on Occupational Health Service. Special issue of Policy and Practice in Health and Safety. Leicester: IOSH Services Ltd; 2007. p. 1-190.

[38] Lehtinen S, Rantanen J, Kim R (2012). National Profile of Occupational Health System in Finland.

[39] Higashi T (2006). Study on a Model for Future Occupational Health: Proposals for an Occupational Health Service Model in Japan. Ind Health.

[40] Rantanen J, Kauppinen T, Toikkanen J, Kurppa K, Lehtinen S, Leino T (2001). Work and health country profiles. Country profiles and national health surveillance indicators in occupational health and safety. People and Work. Research Reports 44.

[41] Rantanen J, Kauppinen T, Lehtinen S, Toikkanen J, Kurppa K, Leino T, Mattila M, editors. Work and health country profiles of twenty-two European countries. People and Work. Research Reports 52. Helsinki: Finnish Institute of Occupational Health; 2002.

[42] Kurppa K, Lehtinen S, editors. Local Occupational Health and Safety Profiles and Indicators. People and Work Research Reports 64. Helsinki: Finnish Institute of Occupational Health; 2004.

[43] Lehtinen S, Kurppa K, Rantanen J, editors. Proceedings of the Workshop on National and Local OH&S Profiles and Indicators. People and Work. Research Reports 55. Helsinki: Finnish Institute of Occupational Health; 2002.

[44] Lehtinen S, Rantanen J, Elgstrand K, Liesivuori J, Peurala M, editors. Challenges to occupational health services in the Regions. The national and international responses. Proceedings of a WHO/ICOH/ILO Workshop, 24 January 2005. Helsinki: Finnish Institute of Occupational Health; 2005.

[45] Elgstrand K, editor. OSH needs in developing countries. OSH & Development. 2010; 10:1-108.

[46] World Health Organization. Occupational health. Country occupational health and safety profiles. http://www.who.int/occupational_health/topics/profiles/en/. Accessed 12 Dec 2016.

[47] Lappalainen K, Aminoff M, Hakulinen H, Hirvonen M, Räsänen K, Sauni R, Stengård J. Työterveyshuolto Suomessa vuonna 2015: ja kehitystrendi 2000–2015. (Occupational health services in Finland in 2015 and trends 2000-2015). Helsinki: Työterveyslaitos ja kirjoittajat; 2016. https://www.julkari.fi/handle/10024/131293. Accessed on 12 Dec 2016.

[48] Mizoue T, Higashi T, Muto T, Yoshimura T, Fukuwatari Y (1996). Activities of an occupational health organization in Japan, in special reference to services for small- and medium-scale enterprises. Occup Med.

[49] Occupational health services: an overview. Rantanen J, editor. WHO Regional Publications. European Series No. 26. Copenhagen: WHO Regional Office for Europe; 1990. 48 p. 2182043

[50] International Labour Organization. Labour Statistics. ILO Laborsta 2008 and 2010. Geneva: ILOSTAT - ILO; 2008, 2010. http:// www.ilo.org/ilostat. Accessed 12 Dec 2016.

[51] International Labour Organization. Convention No. 155 concerning Occupational Safety, Health and the Working Environment. Geneva: International Labour Conference; 67th session. 1981. https://www.ilo.org/dyn/normlex/en/f?p=normlexpub:12100:0::no::p12100_instrument_id:312300. Accessed 13 Dec 2016.

[52] International Labour Organization. Guidelines on occupational safety and health management systems, ILO-OSH 2001. Geneva: ILO; 2001. http://www.ilo.org/wcmsp5/groups/public/-%2D-ed_protect/-%2D-protrav/-%2D-safework/documents/normativeinstrument/wcms_107727.pdf. Accessed 14 Dec 2016.

[53] Siriruttanapruk S (2006). Integrating Occupational health services into Public Health Systems: A Model Developed with Thailand's Primary Care Units. Series Number 2.

[54] Rui C (2010). China’s Experience with Basic Occupational Health Services.

[55] Pingle S, Elgstrand K (2010). OSH needs in developing countries: India. OSH Needs in developing countries.

[56] World Health Organization. WHA62.12 on Primary Health Care. http://www.who.int/hrh/resources/A62_12_EN.pdf. Accessed 14 Dec 2016.

[57] Rantanen J. Basic Occupational health services. Lehtinen S, editor. Helsinki: Finnish Institute of Occupational Health; 2007. 24 p. http://partner.ttl.fi/en/publications/Electronic_publications/Documents/BOHS3Edition28Sept2007_3_.pdf. Accessed 12 Dec 2016.

[58] International Labour Organization. Recommendation No. 171 concerning Occupational health services. Geneva: 71st ILC session, 26 Jun 1985. http://www.ilo.org/dyn/normlex/en/f?p=NORMLEXPUB:12100:0::NO::P12100_INSTRUMENT_ID:312509. Accessed 14 Dec 2016.

[59] International Labour Organization. Occupational health services recommendation No. 112. Geneva: 43rd ILC session, 24 Jun 1959. https://www.ilo.org/dyn/normlex/en/f?p=NORMLEXPUB:12100:0::NO:12100:P12100_INSTRUMENT_ID:312450:NO. Accessed 14 Dec 2016.

[60] WHO Regional office of Europe (1989). Final Report of consultation on Occupational health services, Helsinki 22–24 May 1989. Publication No. ICP/OCH/134.

[61] Taskinen H, editor. Good Occupational Health Practice. A guide for planning and follow-up of occupational health services. Revised 2nd ed. Helsinki: Ministry of Social Affairs and Health, Finnish Institute of Occupational Health; 2004.

[62] Ministry of Social Affairs and Health. National Occupational Safety and Health Profile of Finland. Helsinki: Ministry of Social Affairs and Health; 2006:8. 80 p. http://www.ilo.org/wcmsp5/groups/public/-%2D-ed_protect/-%2D-protrav/-%2D-safework/documents/policy/wcms_179869.pdf . Accessed 15 Dec 2016.

[63] Tu NTH, Anh LM (2009). Occupational health services in Vietnam. Asian-Pacific Newsl Occup Health Saf.

[64] World Health Organization. Description of the Key Informant Survey. 2001. http://www.who.int/responsiveness/surveys/KIS_2001_Methodology.pdf. Accessed 14 Dec 2016.

[65] De Silva A, Valentine N. Measuring responsiveness: Results of a key informants survey in 35 countries. GPE Discussion Paper No. 21. Geneva: WHO; 2000. http://www.who.int/healthinfo/paper21.pdf. Accessed 14 Dec 2016.

[66] Valentine NB, De Silva QA, Murray CJL. Estimating Responsiveness Level and Distribution for 191 Countries: Methods and Results. GPE Discussion Paper Series: No. 22. EIP/GPE/FAR. Geneva: WHO; 2000. http://www.who.int/healthinfo/paper22.pdf . Accessed 14 Dec 2016.

[67] Parsons J A, Baum S, Johnson T P. Inclusion of disabled populations in social surveys: Review and recommendations. Large print version. Chicago, Illinois: National Center for Health Statistics, University of Illinois at Chicago; 2000. http://www.srl.uic.edu/Publist/StdyRpts/838disability/DisabledPops.pdf. Accessed 14 Dec 2016.

[68] International Labour Organization. Committee on Employment and Social Policy. Occupational safety and health: Synergies between security and productivity. Geneva: International Labour Office, GB.295/ESP/3 Governing Body 295th Session March 2006. http://www.ilo.org/wcmsp5/groups/p http://www.srl.uic.edu/Publist/StdyRpts/838disability/DisabledPops.pdf ublic/@ed_protect/@protrav/@safework/documents/meetingdocument/wcms_110380.pdf . Accessed 15 Dec 2016.

[69] Rantanen J, Lehtinen S, Mikheev M, editors. Health Protection and Health Promotion in Small-scale Enterprises. Proceedings of the Joint WHO/ILO Task Group 1–3 November 1993. Helsinki: Finnish Institute of Occupational Health; 1994. 178 p.

[70] World Health Organization. Working for health and growth: investing in the health workforce. Geneva: WHO; 2016. http://www.who.int/hrh/com-heeg/reports/en/. Accessed 16 Dec 2016.

[71] World Health Organization. The World Health Report 2006 - working together for health. Geneva: WHO; 2006. http://www.who.int/whr/2006/en/. Accessed 16 Dec 2016.

[72] Virtanen P, Oksanen T, Kivimäki M, Virtanen M, Pentti J, Vahtera J (2008). Work stress and health in primary health care physicians and hospital physicians. Occup Environ Med.

[73] Wilson DJ, Takahashi K, Sakuragi S, Yoshino M, Hoshuyama T, Imai T, Takala J (2007). The Ratification Status of ILO Conventions Related to Occupational Safety and Health and Its Relationship with Reported Occupational Fatality Rates. J Occup Health.

[74] World Health Organization. Declaration of Alma-Ata. International Conference on Primary Health Care, Alma-Ata, USSR, 6-12 September 1978. Geneva: WHO; 1978. http://www.who.int/publications/almaata_declaration_en.pdf?ua=1. Accessed 16 Dec 2016.

[75] Safety and Health at Work: A Vision for Sustainable Prevention. XX World Congress on Safety and Health at Work. Frankfurt, Germany, 2014. http://www.ilo.org/wcmsp5/groups/public/@ed_protect/@protrav/@safework/documents/publication/wcms_301214.pdf. Accessed 16 Dec 2016.

[76] Australian Government. The Cost of Work-related Injury and Illness for Australian Employers, Workers and the Community: 2005-06. Commonwealth of Australia; 2009. http://safeworkaustralia.gov.au/AboutSafeWorkAustralia/WhatWeDo/Publications/Documents/178/CostsofWorkRelatedInjuryAndDisease_Mar2009.pdf. Accessed 16 Dec 2016.

[77] Hämäläinen P, Saarela K-L, Takala J (2009). Global trend according to estimated number of occupational accidents and fatal work-related diseases at region and country level. J Saf Res.

[78] Takala J, Hämäläinen P, Nenonen N, Takahashi K (2017). Odgerel Chimed-Ochir, Rantanen J. Comparative Analysis of the Burden of Injury and Illness at Work in Selected Countries and Regions. Cent Eur J Occup Environ Med.

[79] Mills PR, Kessler RC, Cooper J. & Sullivan S. Impact of a Health promotion program on Employee health risks and work productivity. Am J Health Promot. 2007;22(1) 10.4278/0890-1171-22.1.45 17894263

[80] EU OSHA. The business benefits of good occupational safety and health. Fact sheet 77/EN. Bilbao, 2007. https://osha.europa.eu/en/tools-and-publications/publications/factsheets/77. Accessed 16 Dec 2016.

[81] ICOH (2016). Statement on the Report of the UN High-Level Commission on Health, Employment and Economic Growth.

[82] Employment Conditions and Health Inequalities Final Report to the WHO Commission on Social Determinants of Health (CSDH) Employment Conditions Knowledge Network (EMCONET). Joan Benach, Carles Muntaner, Vilma Santana (Chairs) Employment Conditions Knowledge Network (EMCONET). Final Report, 20 September 2007. http://www.who.int/social_determinants/resources/articles/emconet_who_report.pdf. Accessed 16 Dec 2016.

