# Supplementary material for: A global survey on occupational health services in selected international commission on occupational health (ICOH) member countries
Source: BMC Public Health. 2017 Oct 5;17:787. doi: 10.1186/s12889-017-4800-z (PMC5629797; doi:10.1186/s12889-017-4800-z)
Supplement: Additional file 1: — Criteria for OHS Index. (DOCX 17 kb) [file 12889_2017_4800_MOESM1_ESM.docx]

**Criteria for OHS Index Additional file 1**

| **Criterion** | **Scale** | **Score for country** |
| --- | --- | --- |

**1. Ratification, Policy, Strategy**

| ILO convention 161 + National OHS Policy & Programme endorsed + implemented | 5 |  |
| --- | --- | --- |
| Not ratified but principles of ILO Instruments C 161 and R 171 and WHO GPA used as guidance and National OHS Programme drawn up + implemented widely | 4 |  |
| National Policy, Strategy and Programme available and implemented | 3 |  |
| Limited scope for OHS Programme (selected sectors or other target groups) | 2 |  |
| No formally approved Policy, Strategy or Programme nor reference to ILO or other international instruments in national policy | 1 |  |

**2. Legislation for OHS**

| Special OHS law available and implemented widely | 5 |  |
| --- | --- | --- |
| Provisions for OHS in other legislations e.g. OSH law and implemented widely | 4 |  |
| Limited obligations for the employers for organization OHS (e.g. size limits for companies or only for high risk groups) | 3 |  |
| Collective agreement by social partners on OHS | 2 |  |
| Totally voluntary OHS | 1 |  |

**3. Coverage of OHS (i.e. access to services in everyday practice, not just legal text)**

| Coverage of total employed population 80-100% | 5 |  |
| --- | --- | --- |
| Coverage of total employed population 60-79% | 4 |  |
| Coverage of total employed population 30-59 | 3 |  |
| Coverage of total employed population 10-29% | 2 |  |
| No organized government governance below 10% | 1 |  |

**4. Support services at secondary and tertiary levels**

| Well organized and institutionalized multidisciplinary support services available universally at national and provincial/regional /district levels | 5 |  |
| --- | --- | --- |
| Well organized institutionalized multidisciplinary support services at national but not universally at provincial/regional /district levels | 4 |  |
| Sporadic support services by public or private organizations (e.g. consultancies) | 3 |  |
| Support available on limited disciplines e.g. occupational medicine only | 2 |  |
| No organized system for support services | 1 |  |

**5. Density of occupational health physicians (in service provision at grassroots level)**

| Density of OHPs 1/1000-1/2000 | 5 |  |
| --- | --- | --- |
| Density of OHPs 1/2001-1/5000 | 4 |  |
| Density of OHPs 1/5001-1/7500 | 3 |  |
| Density of OHPs 1/7501- 10000 | 2 |  |
| Density of OHPs lower than 1/10000 | 1 |  |

**6. Multidisciplinarity of OHS staffs**

| 5-6 specialists e.g. OHP, OHN, Occupational hygienist, Psychologist, Ergonomist, Safety engineer or OD expert available widely throughout whole OHS system | 5 |  |
| --- | --- | --- |
| 3-4 specialists including OHP and OHN widely available | 4 |  |
| OHP and OHN | 3 |  |
| OHP only | 2 |  |
| No experts trained in OH | 1 |  |

**7. Content and activities of OHS (in practical service provision, not just in regulations)**

| Comprehensive service with prevention of accidents and diseases and first aid, risk assessment, promotion of health and work ability, curative services, development of work organization, rehabilitation & return to work (RTW) | 5 |  |
| --- | --- | --- |
| Preventive and limited other activities | 4 |  |
| Prevention only | 3 |  |
| Health examinations and curative activities only | 2 |  |
| Health examinations only | 1 |  |

**8. Financing for sustainable OHS system**

| Legislation-based financing with employers primary responsibility and/or pooling their contributions through insurance and public financing for non-contributors | 5 |  |
| --- | --- | --- |
| Legislation-based financing for organized sectors only (directly by employer or through insurance) | 4 |  |
| Financing from public sources only without contribution of employers | 3 |  |
| Voluntary employer or insurance financing | 2 |  |
| Sporadic financing by the employer | 1 |  |
